# Divergence-Based Risk Measures: A Discussion on Sensitivities and Extensions

**DOI:** 10.3390/e21070634

**Published:** 2019-06-27

**Authors:** Meng Xu, José M. Angulo

**Affiliations:** 1 School of Economics, Sichuan University, Chengdu 610065, China; 2 Department of Statistics and Operations Research, University of Granada, 18071 Granada, Spain

**Keywords:** convex risk measure, preference, sensitivity analysis, ambiguity, *ϕ*-divergence

## Abstract

This paper introduces a new family of the convex divergence-based risk measure by specifying (h,ϕ)-divergence, corresponding with the dual representation. First, the sensitivity characteristics of the modified divergence risk measure with respect to profit and loss (P&L) and the reference probability in the penalty term are discussed, in view of the certainty equivalent and robust statistics. Secondly, a similar sensitivity property of (h,ϕ)-divergence risk measure with respect to P&L is shown, and boundedness by the analytic risk measure is proved. Numerical studies designed for Rényi- and Tsallis-divergence risk measure are provided. This new family integrates a wide spectrum of divergence risk measures and relates to divergence preferences.

## 1. Introduction

In the last two decades, there has been a substantial development of a well-founded risk measure theory, particularly propelled since the axiomatic approach introduced by [1] in relation to the concept of coherency. While, to a large extent, the theory has been fundamentally inspired and motivated with financial risk assessment objectives in perspective, many other areas of application are currently or potentially benefited by the formal mathematical construction of the discipline.

The coherency axioms of monotonicity, translation invariance, positive homogeneity and sub-additivity lead to a representation for coherent risk measures of the form

(1) $$\rho \left(X\right)=\underset{Q\in Q}{\sup}E_{Q}\left[-X\right],$$

where *X* is a real-valued measurable function on the measurable space (Ω,F) representing benefit, and Q is a certain set of probability measures on (Ω,F). Since its introduction, such a formulation has been the matter of an important debate in the following years, mainly due to the restrictive conditions implied by the axiom of sub-additivity, which make coherent measures of risk unsuitable in certain applications. Relaxation of this axiom in terms of a weaker convexity condition, as was introduced by [2,3], provides a more flexible representation in the form

(2) $$\rho \left(X\right)=\underset{Q\in Q}{\sup}\left\{E_{Q}\left[-X\right]-\alpha \left(Q\right)\right\},$$

where Q is a suitable set of probability measures on (Ω,F), and α is a penalty function defined on Q. The minimal penalty function for ρ is convex, being defined by

$$\alpha_{min}\left(Q\right):=\underset{X\in X}{\sup}\left\{E_{Q}\left[-X\right]-\rho \left(X\right)\right\},$$

where X is the space of all Borel-measurable functions defined on (Ω,F). With additional assumptions (see, for example, [4]), there exists a one-to-one pairing between ρ and $\alpha_{min}$ via Legendre-Fenchel (LF) duality. The theories of convex and coherent risk measures have been increasingly and deeply developed as a central axis of the general theory of measures of risk, owing to the contributions by many researchers (e.g., [4,5,6,7,8,9,10,11,12], etc; see also early work of [13], among others). In the law-invariant case [14], the value ρ(X) only depends on the distribution of X∈X under the assumption of a prefixed probability measure *P* on (Ω,F). Typical examples are value-at-risk (VaR), average value-at-risk (AVaR), tail value-at-risk (TVaR), the entropic risk measure, the ϕ-divergence risk measure related to the optimized certainty equivalent (OCE), etc; the authors of [15] comparatively study various generalized entropy-based forms of risk measures. The details on the VaR related family of risk measures can be found in the large range of the literature connected with duality. Here we just review some basic definitions focused on the entropic risk measure and ϕ-divergence risk measure used in the examples of Section 2.1.

In the decision theory under uncertainty, especially in the context of economics, the numerical representation of preferences shares a similar structure as the dual form of risk measures. In this context, it is postulated that an agent can evaluate the consequences of an alternative decision according to

(3) $$U\left(X\right)=\underset{Q\in Q}{\inf}\left\{E_{Q}\left[u\left(X\right)\right]+\alpha \left(Q\right)\right\},$$

where *u* denotes the utility function and the penalty function α here is explained as the ambiguity attitude in this related strand of the literature. For more details on the ambiguity of variational preferences, we refer to the work by [16], which also proves that the preference relation satisfies the axioms of preferences when there exists a non-constant affine utility function and demonstrates a new class of preferences, built on the ϕ-divergence, for handling Ellsberg-type puzzles [17]. This numerical form leads to the definition of a loss functional, L(X)=−U(X), satisfying

$$L\left(X\right)=\underset{Q\in Q}{\sup}\left\{E_{Q}\left[-u\left(X\right)\right]-\alpha \left(Q\right)\right\},$$

but we are interested in the risk measure, the payoff of the financial position, which is equivalent to the negative certainty equivalent

$$\underset{Q\in Q}{\sup}\left\{E_{Q}\left[\varphi \left(-X\right)\right]-\alpha \left(Q\right)\right\},$$

where φ(x)=−u(−x), named as the de-utility function. The (generalized) Donsker-Varadhan variational formula [18,19] plays a vital role in these connections, which recently also spread to the classical Gini index of income inequality [20] through the linkage tool derived from [21]. Some other efforts have been made to connect both of these research fields, such as in [2,7], and more recently [22].

From the perspective of convex risk measures, intuitively, the dual representation of Equation (2) can be regarded as a risk-measure generator obeying the axiomatic framework, i.e., we are able to produce a certain risk measure through selecting among different penalty functions. For instance, [8] constructed a bridge between the expected utility framework and the ϕ-divergence risk-measure framework (Lemma A2). On the one hand, any utility function satisfying certain suitable conditions can generate its related convex risk measure. Conversely, any ϕ-divergence can provide a possibly analytic form of a risk measure useful for calculation in practice, such as the Tsallis divergence.

Thus, the penalty function plays a substantive role in the evaluation of risk, with its properties determining the behavior of the linked risk measure. Moreover, from our understanding, this viewpoint on the sensitivity is ignored in the dual theory of convex risk measures. Besides early work, as in [23,24], discussing the sensitivity concentrated on cases of VaR-type with respect to portfolio allocation, recently, the authors of [25] have stated a framework for sensitivity analysis both on the measurement processes and the dataset through robust statistics and the estimation procedures, respectively. These sensitivity studies focus on trend identification (direction of change), which reveals how a shift of position affects the output risk measurements. In an extensive review, [26] identify and comparatively discuss different sensitivity analysis strategies in the literature, with a clear distinction between local (deterministic) and global (probabilistic) approaches.

The contribution of this paper is twofold. First, after reviewing the literature on the ambiguity aversion (or loving) of preferences in Section 2.2, we study the sensitivity of convex risk measures and examine the modified version of the ϕ-divergence risk measure to declare this issue clearly and operationally presented in Section 2.2 and Section 3 in terms of the reference probability measure and the input financial position.

Moreover, there are some important measures of divergence that cannot be obtained as particular cases of the ϕ-divergence; for instance, Rényi divergence. For this reason, the authors of [27] worked with an extended formulation, the so-called (h,ϕ)-divergence, denoted by $D_{\phi }^{h}\left(Q,P\right)$, from which various well-known divergences can be obtained through the extra distortion *h*. Our second contribution is, in a first step, straightforward: By replacing the penalty function to the (h,ϕ)-divergence in the dual form of convex risk measure, we derive a new family of convex risk measures, the (h,ϕ)-divergence risk measure

$$\rho_{h}\left(X\right):=\underset{Q\in Q}{\sup}\left\{E_{Q}\left[-X\right]-D_{\phi }^{h}\left(Q,P\right)\right\}.$$

Recent study on risk measures has focused on the extension of the risk-neutral valuation. Under the probability measure *Q*, the part $E_{Q}\left[-X\right]$ is equivalent to the neutral risk. One more general approach to evaluating *X* consists in considering $c_{\varphi }\left(X,Q\right)=\varphi^{-1}E_{Q}\left[\varphi \left(-X\right)\right]$, where a non-linear and convex φ leads to a risk-averse evaluation by the so-called φ-convex risk measures. Based on this extension, the authors of [7] develop a subclass of the entropic risk measure and contribute the connections with variational preferences by assuring the de-utility function φ as linear or exponential. The authors of [9] extend to a more general form based on the utility theory, while the authors of [28] develop the optimal expected utility (OEU) risk measure by modifying the OCE, which benefits from the easy application defined on optimizing in the real field.

Our article is organized as follows. In Section 2, we briefly review the content of the dual representation for convex risk measures and the concept of ambiguity in the field of the preference in decision making theory. In Section 3, we strengthen the analysis on the modified version of ϕ-divergence risk measure and its sensitivity on the financial positions, and analyze the sensitivity on the probability measure. In Section 4, we extend the penalty term to the (h,ϕ)-divergence for defining the new family of convex risk measures, and derive relative OCE bounds. The case of Rényi divergence is addressed in particular. Some numerical studies by cases are shown in Section 5. Conclusions and directions of continuing work are given in Section 6.

## 2. Risk Measures and Ambiguity

There is a deep connection between the concept and representation of risk measure and ambiguity. In the next subsection, we review several definitions and special cases of a measure of risk in view of axioms and duality, and illustrate its interpretation associated with the structure of the penalty term in relation to the ambiguity based on the background of the decision making theory in Section 2.2.

### 2.1. Dual Representation of Risk Measures

Let Ω be a fixed set of scenarios. A financial position is typically uncertain, and modeled as a real-valued measurable function *X* on the measurable space (Ω,F), for a given σ−algebra F.

**Definition** **1.** *ρ:X→R is called a ’convex measure of risk’ if it satisfies the following conditions for all X,Y∈X:*

*1.*  *Convexity: ρ(λX+(1−λ)Y)≤λρ(X)+(1−λ)ρ(Y), for λ∈[0,1].*
*2.*  *Monotonicity: If X≤Y, then ρ(X)≥ρ(Y).*
*3.*  *Translation Invariance: If m∈R, then ρ(X+m)=ρ(X)−m.*

*A convex measure of risk ρ is called a ’coherent measure of risk’ if it meets the property of*

*4.*  *Positive Homogeneity: If λ≥0, then ρ(λX)=λρ(X).*

Following the robust representation of convex measures of risk in [2], we recall some notations. $M_{1}:=M_{1}\left(\Omega ,F\right)$ denotes the class of all probability measures on (Ω,F), and $M_{1,f}:=M_{1,f}\left(\Omega ,F\right)$ denotes the class of all finitely additive and non-negative set functions *Q* on F which are normalized to Q[Ω]=1. Let $\alpha :M_{1,f}\to R\cup \left\{+\infty \right\}$ be any functional which is bounded from below and not identically equal to +∞. For each $Q\in M_{1,f}$ the functional $X\to E_{Q}\left[-X\right]-\alpha \left(Q\right)$ is convex, monotone, and translation invariant, and these three properties are preserved when taking the supremum over Q∈Q. Hence,

$$\rho \left(X\right):=\underset{Q\in M_{1,f}}{\sup}\left\{E_{Q}\left[-X\right]-\alpha \left(Q\right)\right\}$$

defines a dual form of a convex measure of risk on X.

We recall the definitions of entropic risk measure and the ϕ-divergence risk measure (named *g*-divergence in [8]), which motivate our contributions.

**Entropic risk measure.** The standard ’entropic risk measure’ is defined by

(4) $$e_{\gamma }\left(X\right):=\frac{1}{\gamma }\ln E_{P}\left[e^{-\gamma X}\right],$$

for parameter γ∈[0,∞). Its dual representation is given by

(5) $$e_{\gamma }\left(X\right)=\underset{Q\in Q}{\sup}\left\{E_{Q}\left[-X\right]-\frac{1}{\gamma }D\left(Q,P\right)\right\},$$

where D(Q,P) denotes the relative entropy or Kullback-Leibler (KL) divergence of *Q* with respect to *P*.

When D(Q,P)≤d, for a constant *d*, the entropic risk measure is coherent [6]. The entropic risk measure is related to Varadhan’s Lemma (Lemma A3) in the large deviation theory, when the rate function is the relative entropy.

**ϕ-divergence risk measure.** A natural extension can be formulated as the ’ϕ-divergence risk measure’ in terms of a ϕ-divergence $D_{\phi }\left(Q,P\right)$,

(6) $$\rho_{\phi }\left(X\right)=\underset{Q\in Q}{\sup}\left\{E_{Q}\left[-X\right]-D_{\phi }\left(Q,P\right)\right\},$$

with ϕ being a convex function satisfying certain suitable conditions.

**Remark** **1.** *The ‘optimized certainty equivalent’ (OCE) representation by [8,19] can be derived from the generalized Donsker-Varadhan variational formula, as follows:*

(7) $$\begin{array}{ccc} \rho_{OCE}\left(X\right) & = & -\underset{\eta \in R}{\sup}\left\{\eta -E_{P}\left[\phi^{*}\left(\eta -X\right)\right]\right\} \end{array}$$

(8) $$\begin{array}{cc} = & \underset{Q\in Q}{\sup}\left\{E_{Q}\left[-X\right]-D_{\phi }\left(Q,P\right)\right\}, \end{array}$$

where $u\left(\cdot \right)=-\phi^{*}\left(-\cdot \right)$ denotes the utility function, with $\phi^{*}$ being the conjugate of ϕ.

**Remark** **2.** *The OCE representation of Equation (7), referring to Kusuoka representation (see also [29,30]) in case of law invariance, is interpreted as the optimal decision of the allocation of X between the present η and the future consumption.*

For various choices of ϕ corresponding to different divergences, we refer to [31] and applications in [15]. Since Tsallis divergence is an element in the family of ϕ-divergence, we can plug it into Equation (6) to achieve the Tsallis-divergence risk measure $\rho_{T}\left(X\right)$, with $\phi \left(x\right)=\left(x^{1-\alpha }-1\right)/\left(\alpha -1\right)$ for α∈(0,∞).

**Average value-at-risk (AVaR).** The average value-at-risk (AVaR) is defined, for α∈(0,1), as

$${\mathrm{AVaR}}_{\alpha }\left(X\right)=\frac{1}{\alpha }\int_{0}^{\alpha }{\mathrm{VaR}}_{p}\left(X\right)\;dp,$$

where VaR denotes value-at-risk, ${\mathrm{VaR}}_{\alpha }\left(X\right)=\inf\left\{m\in R:P\left[m+X<0\right]\leq \alpha \right\}$, and it can be rewritten into the LF-dual and the OCE-type forms as

(9) $${\mathrm{AVaR}}_{\alpha }\left(X\right)=\underset{Q\in Q_{\alpha }}{\sup}E_{Q}\left[-X\right]=-\underset{\eta \in R}{\sup}\left\{\eta -\frac{1}{\alpha }E_{P}\left[{\left(\eta -X\right)}_{+}\right]\right\},$$

where $Q_{\alpha }$ is the set of all probability measures Q≪P whose density dQ/dP is *P*-a.s. bounded by 1/α. Here, for any z∈R, we denote ${\left(z\right)}_{+}=\max\left\{0,z\right\}$.

### 2.2. Ambiguity

In the language of preference, the penalty term in the dual form of a convex measure of risk can be interpreted as the ambiguity aversion. Here we give some information on the ambiguity attitudes characterized by the variational preferences and the original framework followed by [16,32], respectively. The preference denoted by ≿ is called ’variational’ if and only if there exists a non-constant affine function u:X→R and a grounded, convex and lower semicontinuous function α such that, for all acts $X_{1}$, $X_{2}\in X$,

(10) $$\begin{array}{ccc} X_{1}≿X_{2} & \Leftrightarrow & \underset{Q\in Q}{\inf}\left\{E_{Q}\left[u\left(X_{1}\right)\right]+\alpha \left(Q\right)\right\}\geq \underset{Q\in Q}{\inf}\left\{E_{Q}\left[u\left(X_{2}\right)\right]+\alpha \left(Q\right)\right\} \end{array}$$

(11) $$\begin{array}{cc} \Leftrightarrow & \rho \left(u\left(X_{1}\right)\right)\leq \rho \left(u\left(X_{2}\right)\right). \end{array}$$

For each *u* there is a (unique) minimal $\alpha_{min}$ that satisfies Equation (10), given by

$$\alpha_{min}\left(Q\right)=\underset{X\in X}{\sup}\left\{E_{Q}\left[-u\left(X\right)\right]-\rho \left(u\left(X\right)\right)\right\}=\underset{X\in X}{\sup}\left\{E_{Q}\left[-u\left(X\right)\right]+u\left(x_{X}\right)\right\},$$

where $x_{X}$ is a ‘certainty equivalent’ for *X* (see [16]).

According to [32], the benchmark preference, subjective expected utility (SEU) preference, is introduced to comparatively judge the ambiguity neutrality (see also [33]), and is quantified by [34] via the Arrow-Pratt quadratic approximation. All the variational preferences are ambiguity-averse. In order to distinguish the ambiguity attitudes among the variational preferences, the penalty function α gets a contextually substantive interpretation in the following result. Note that $u_{1}\approx u_{2}$ means that there exist a positive constant $d_{1}$ and a constant $d_{2}$ such that $u_{1}=d_{1}u_{2}+d_{2}$.

**Proposition** **1** (Proposition 8 in [16])**.** *Given two variational preferences ${≿}_{1}$ and ${≿}_{2}$, the relation ${≿}_{1}$ is more ambiguity-averse than ${≿}_{2}$ if and only if $u_{1}\approx u_{2}$ and $\alpha_{min,1}\leq \alpha_{min,2}$, provided that $u_{1}=u_{2}$.*

By the assumption of common normalization, $u_{1}=u_{2}$, thus, the more ambiguity-averse results from the smaller penalty $\alpha_{min}$, and $\alpha_{min}$ is interpreted as the ‘index of ambiguity’ in agreement with the penalty term in the convex risk measure.

## 3. Properties of Divergence Risk Measure

### 3.1. Modified ϕ-Divergence Risk Measure

The modified ϕ-divergence risk measure, involving a linear rescaling of the penalty term, is defined below.

**Definition** **2.** *The modified ϕ-divergence risk measure with parameter θ∈R is formulated as*

(12) $$\rho_{M}\left(X\right):=\underset{Q\in Q}{\sup}\left\{E_{Q}\left[-X\right]-\theta D_{\phi }\left(Q,P\right)\right\},$$

*where $D_{\phi }\left(Q,P\right)$ is the ϕ-divergence.*

The formulation above comes from the divergence preference approach in [16] (see also [19]), where a slightly more general case of weighted ϕ-divergence is considered. Compared to the ϕ-divergence risk measure of Equation (8), it benefits from the extra parameter θ, which can be seen as an indicator to control the weight of the penalty, i.e., the relative importance of ambiguity aversion in the construction of the risk measure. It represents the same role as 1/γ in the standard entropic risk measure of Equation (4). The Donsker-Varadhan variational formula can be rewritten for these cases as follows.

**Proposition** **2.** *For parameter $\theta \in R^{+}$,*

$$\underset{Q\in Q}{\inf}\left\{E_{Q}\left[X\right]+\theta D_{\phi }\left(Q,P\right)\right\}=\underset{\eta \in R}{\sup}\left\{\eta -E_{P}\phi_{\theta }^{*}\left(\eta -X\right)\right\},$$

with $\phi_{\theta }^{*}\left(\cdot \right)$ being the Legendre-Fenchel transform of θ·ϕ(·)

$$\phi_{\theta }^{*}\left(x\right)=\underset{t\in \mathrm{dom}\phi }{\sup}\left\{t\cdot x-\theta \cdot \phi \left(t\right)\right\}.$$

Correspondingly, the modified ϕ-divergence risk measure can be also rewritten by the following OCE-type form:

(13) $$\rho_{M}\left(X\right)=-\underset{\eta \in R}{\sup}\left\{\eta -E_{P}\phi_{\theta }^{*}\left(\eta -X\right)\right\}.$$

The parameter θ calibrates the relative effect of penalization in terms of the discrepancy of the measure *Q* with respect to the reference measure *P*. The limiting cases where θ tends to 0 or *∞* are shown in the next result.

**Proposition** **3** (Proposition 22 in [16])**.** *The following limiting cases for θ hold:*

*(1)*  $\lim_{\theta ↓0}\sup_{Q\in M_{1,f}\left(\Omega \right)}\left\{E_{Q}\left[-X\right]-\theta D_{\phi }\left(Q,P\right)\right\}=-{ess inf}_{\omega }X\left(\omega \right)$.
*(2)*  $\lim_{\theta ↑\infty }\sup_{Q\in M_{1,f}\left(\Omega \right)}\left\{E_{Q}\left[-X\right]-\theta D_{\phi }\left(Q,P\right)\right\}=E_{P}\left[-X\right]$.

It is understood in the context of preferences that agents that behave by means of the criterion are assuming that the reference *P* may not be the right probability measure to reflect their interests and a potential probability measure *Q* may be taken into consideration, weighted by the parameter θ. The larger the value of θ, the higher the credibility attributed to *P* as the correct model.

### 3.2. Sensitivity Analysis with Respect to *X*

We first analyze the static sensitivity of the modified risk measure in terms of the financial position *X* at the direction towards $X^{\prime }$ in the space X. For convenience regarding the description below, we introduce some notation. Let $f_{Q}\left(X\right):=E_{Q}\left[-X\right]-\theta D_{\phi }\left(Q,P\right)$. The modified ϕ-divergence risk measure can be rewritten as $\rho_{M}\left(X\right)=\sup_{Q}f_{Q}\left(X\right)$. It is natural to define Gâteaux differentiability ([35], Chapter 2) to describe the derivative in the direction $X^{\prime }-X$, that is,

$${\left.\frac{\partial }{\partial r}\rho_{M}\left(X_{r}\right)\right|}_{r=0}=\lim_{r\to 0}\frac{\rho_{M}\left(X_{r}\right)-\rho_{M}\left(X\right)}{r},$$

where $X_{r}$ denotes the intermediate position at the direction towards $X^{\prime }$ given by

$$X_{r}=\left(1-r\right)X+rX^{\prime }.$$

According to [7,16], we can derive the following proposition.

**Proposition** **4.** *The modified ϕ-divergence risk measure is everywhere differentiable for all θ>0, and, assuming that $Q_{0}=\arg\sup_{Q}f_{Q}\left(X\right)$ exists,*

(14) $${\left.\frac{\partial }{\partial r}\rho_{M}\left(X_{r}\right)\right|}_{r=0}=\lim_{r\to 0}\frac{\rho_{M}\left(X_{r}\right)-\rho_{M}\left(X\right)}{r}=E_{Q_{0}}\left[X-X^{\prime }\right].$$

**Remark** **3.** *See, for example, [4,36] for conditions ensuring the existence of $Q_{0}$.*

**Proof.**  At any certain point *X* for the financial position, in the direction towards $X^{\prime }$, given $Q_{0}=\arg\sup_{Q}f_{Q}\left(X\right)$, we have

$$\begin{array}{ccc} {\left.\frac{\partial }{\partial r}\rho_{M}\left(X_{r}\right)\right|}_{r=0} & = & \lim_{r\to 0}\frac{\rho_{M}\left(X_{r}\right)-\rho_{M}\left(X\right)}{r}\\ & = & \lim_{r\to 0}\frac{\rho_{M}\left(X_{r}\right)-f_{Q_{0}}\left(X_{r}\right)}{r}+\lim_{r\to 0}\frac{f_{Q_{0}}\left(X_{r}\right)-\rho_{M}\left(X\right)}{r}\\ & = & \lim_{r\to 0}\frac{\rho_{M}\left(X_{r}\right)-f_{Q_{0}}\left(X_{r}\right)}{r}+E_{Q_{0}}\left[X-X^{\prime }\right]. \end{array}$$

Clearly

$$\lim_{r↓0}\frac{\rho_{M}\left(X_{r}\right)-f_{Q_{0}}\left(X_{r}\right)}{r}\geq 0,\;\lim_{r↑0}\frac{\rho_{M}\left(X_{r}\right)-f_{Q_{0}}\left(X_{r}\right)}{r}\leq 0,$$

and by the convexity assumption it follows that

$$\lim_{r\to 0}\frac{\rho_{M}\left(X_{r}\right)-f_{Q_{0}}\left(X_{r}\right)}{r}=0,$$

which completes the proof. □

The above property of sensitivity is only suitable for Gâteaux-differentiable risk measures, whereas more recently the athours of [37] consider the convex risk measures in non-Gâteaux-differentiable cases by means of the Aumann-Shapley allocation principle [38] in a view of capital allocation.

### 3.3. Sensitivity Analysis with Respect to *P*

Apart from the qualitative robustness analysis addressed in [25], which gives an entire systematic framework on examining Hampel’s robustness of risk estimators, the following content shows one aspect of sensitivity by theoretically deriving the error of the risk measure based on Gâteaux differentiability, considering that the reference probability measure *P* has a slight change in the direction towards a certain probability measure $P^{\prime }$ in the space $M_{1}$. The directional derivative can be seen as a measure for the sensitivity of ρ(X) with respect to considering the mixture measure $P_{\gamma }=\left(1-\gamma \right)P+\gamma P^{\prime }$ of *P* and $P^{\prime }$, as γ tends to 0.

For convenience, we adopt the following notation in this section. Let $g\left(\eta ,P\right):=\eta -E_{P}\left[\phi_{\theta }^{*}\left(\eta -X\right)\right]$. Hence, we rewrite

$$\rho_{M}\left(X\right):=\rho \left(X,P\right)=\underset{\eta }{\sup}g\left(\eta ,P\right)=g\left(\eta \left(P\right),P\right),$$

with $\eta \left(P\right)=\arg\sup_{\eta }g\left(\eta ,P\right)$. In what follows, *q* denotes the argument of $\phi_{\theta }^{*}\left(\cdot \right)$.

As before, it is plausible to define the derivative of $\rho (X,P_{\gamma })$ at γ=0 for describing the degree of robustness of the modified ϕ-divergence risk measure, which reflects the effect on $\rho_{M}\left(X\right)$ of a small change of the probability measure *P* in the direction towards the probability measure $P^{\prime }$.

The analysis is then addressed to assess the derivative

(15) $${\left.\frac{\partial }{\partial \gamma }\rho \left(X,P_{\gamma }\right)\right|}_{\gamma =0}=\lim_{\gamma ↓0}\frac{\rho \left(X,P_{\gamma }\right)-\rho \left(X,P\right)}{\gamma }=\lim_{\gamma ↓0}\frac{g\left(\eta \left(P_{\gamma }\right),P_{\gamma }\right)-g\left(\eta \left(P\right),P\right)}{\gamma },$$

where, as stated above, $P_{\gamma }=\left(1-\gamma \right)P+\gamma P^{\prime }$. Observing the OCE-type form of the divergence risk measure (Equation (13)), it is natural to connect with the theories of robust statistics, that is, the influence function referring to [35]. The following lemma discusses the marginal property of η with respect to *P*, where we write $\dot{\eta }$ for short,

$$\dot{\eta }=\lim_{\gamma \to 0}\frac{\eta \left(P_{\gamma }\right)-\eta \left(P\right)}{\gamma }.$$

**Lemma** **1.** Suppose that $\phi_{\theta }^{*}\left(\cdot \right)$ is a second-order differentiable function and $\int_{\Omega }\left(\frac{\partial^{2}}{\partial q^{2}}\phi_{\theta }^{*}\right)\left(\eta \left(P\right)-X\right)\;dP\neq 0$. Then,

(16) $$\dot{\eta }=\frac{1-\int_{\Omega }\left(\frac{\partial }{\partial q}\phi_{\theta }^{*}\right)\left(\eta \left(P\right)-X\right)\;dP^{\prime }}{\int_{\Omega }\left(\frac{\partial^{2}}{\partial q^{2}}\phi_{\theta }^{*}\right)\left(\eta \left(P\right)-X\right)\;dP}.$$

**Proof.**  By optimization in the OCE-type form, we trivially have the relationship between η and the probability measure *P*. For the supremum η with respect to *P*, η(P), we get

$$\frac{\partial }{\partial \eta }g_{\eta }\left(P\right)=0$$

(17) $$\Rightarrow \int_{\Omega }\left(\frac{\partial }{\partial q}\phi_{\theta }^{*}\right)\left(\eta \left(P\right)-X\right)\;dP=1$$

Inserting $P_{\gamma }$ as *P* in the above equation, and taking the derivative on both sides with respect to γ at γ=0, it follows that

$$\dot{\eta }\int \frac{\partial^{2}}{\partial q^{2}}\phi_{\theta }^{*}\left(X-\eta \right)\;dP-\int \frac{\partial }{\partial q}\phi_{\theta }^{*}\left(X-\eta \right)\;dP+\int \frac{\partial }{\partial q}\phi_{\theta }^{*}\left(X-\eta \right)\;dP^{\prime }=0$$

leading to the result. □

Thus, according to Lemma 1, the influence function of the modified ϕ-divergence risk measure can be constructed by substituting Equation (16) into Equation (15).

**Theorem** **1.** *Under the same assumptions of Lemma 1, we have*

$$\begin{array}{ccc} {\left.\frac{\partial }{\partial \gamma }\rho_{M}\left(X\right)\right|}_{\gamma =0} & = & \int_{\Omega }\phi_{\theta }^{*}\left(\eta \left(P\right)-X\right)\;\left(dP^{\prime }-dP\right)\\ & = & E_{P^{\prime }}\left[\phi_{\theta }^{*}\left(\eta \left(P\right)-X\right)\right]-E_{P}\left[\phi_{\theta }^{*}\left(\eta \left(P\right)-X\right)\right]. \end{array}$$

In [25], the influence function is described as the degree of sensitivity function in order to evaluate the level of Hampel’s robustness, which is equivalent to the continuity of the risk measure, and is evolved to the index of qualitative robustness between Hampel’s robustness and full tail sensitivity according to [39,40].

## 4. (h,ϕ)-Divergence Risk Measure

Our new extended family of risk measures derives from (h,ϕ)-divergence, the definition of which in [27] is recalled below.

**Definition** **3.** *An extension of the ϕ-divergence, called (h,ϕ)-divergence, is defined by*

(18) $$D_{\phi }^{h}\left(Q,P\right)=\sum_{a=1}^{A}w_{a}h_{a}\left(\int \phi_{a}\left(\frac{dQ}{dP}\right)dP\right),$$

*for Q≪P, where $h={\left(h_{a}\right)}_{a=1,\cdots ,A}$ and, for a=1,⋯,A, $h_{a}$ are nondecreasing and continuous functions with h(0)=0, $w_{a}$ are positive weights, and the functions $\phi_{a}$ satisfy the conditions for a ϕ-divergence risk measure.*

We denote by Q the set of probability measures *Q* on (Ω,F) which are absolutely continuous with respect to *P*. For simplicity, here we consider the reduced form of the (h,ϕ)-divergence for the case A=1, given by

(19) $$D_{\phi }^{h}\left(Q,P\right)=h\left(\int_{\Omega }\phi \left(\frac{dQ}{dP}\right)\;dP\right).$$

The parameter θ in the modified ϕ-divergence risk measure can be interpreted as the simple case of a linear scale distortion on the ϕ-divergence, whereas the operator *h* is extendedly interpreted as a general case of a non-linear distortion on it.

**Definition** **4.** *Suppose that $D_{\phi }^{h}\left(\cdot ,P\right)$ is convex. The (h,ϕ)-divergence risk measure is defined by*

(20) $$\rho_{h}\left(X\right)=\underset{Q\in Q}{\sup}\left\{E_{Q}\left[-X\right]-D_{\phi }^{h}\left(Q,P\right)\right\}.$$

By the definition of a convex measure of risk and the convexity of the penalty term in the dual form, it is clear to state that the (h,ϕ)-divergence risk measure is a convex measure of risk. It retrieves to the modified ϕ-divergence risk measure in Equation (12) when *h* is linear.

Observing the similar structure to the modified ϕ-divergence risk measure, the following property on the static sensitivity with respect to *X* still holds.

**Proposition** **5.** *Under the same assumptions and setting as in Proposition 4, and assuming that $Q_{0}^{\prime }=\arg\sup_{Q\in Q}\left\{E_{Q}\left[-X\right]-D_{\phi }^{h}\left(Q,P\right)\right\}$ exists,*

(21) $${\left.\frac{\partial }{\partial r}\rho_{h}\left(X_{r}\right)\right|}_{r=0}=\lim_{r\to 0}\frac{\rho_{h}\left(X_{r}\right)-\rho_{h}\left(X\right)}{r}=E_{Q_{0}^{\prime }}\left[X-X^{\prime }\right].$$

The proof can also be derived from [7,16].

The following theorem shows that, under certain conditions, the (h,ϕ)-divergence risk measure can be bounded in terms of the OCE-type form provided by the corresponding (h∘ϕ)-divergence risk measure.

**Theorem** **2.** *Under the convexity of $D_{\phi }^{h}\left(\cdot ,P\right)$, and assuming that $\phi_{h}:=h\circ \phi$ satisfies the conditions for a ϕ-divergence, if h is concave,*

$$\rho_{h}\left(X\right)\leq \rho_{OCE}^{h}\left(X\right);$$

*conversely, if h is convex,*

$$\rho_{h}\left(X\right)\geq \rho_{OCE}^{h}\left(X\right),$$

*with*

$$\rho_{OCE}^{h}\left(X\right)=\underset{Q\in Q}{\sup}\left\{E_{Q}\left[-X\right]-D_{\phi_{h}}\left(Q,P\right)\right\}=-\underset{\eta \in R}{\sup}\left\{\eta -E_{P}\phi_{h}^{*}\left(\eta -X\right)\right\},$$

where $\phi_{h}^{*}$ is the conjugate function of $\phi_{h}$.

**Proof.**  By definition

$$\begin{array}{ccc} \rho_{h}\left(X\right) & = & \underset{Q\in Q}{\sup}\left\{E_{Q}\left[-X\right]-D_{\phi }^{h}\left(Q,P\right)\right\},\;\mathrm{or}\\ -\rho_{h}\left(X\right) & = & \underset{Q\in Q}{\inf}\left\{E_{Q}\left[X\right]+D_{\phi }^{h}\left(Q,P\right)\right\}. \end{array}$$

We denote by *v* the optimal value of the right hand side minimization problem of the above equation (the notations are presented in Appendix A.1):

$$\begin{array}{ccc} v & = & \underset{z\in L^{p}}{\inf}\left\{h\left(\int_{\Omega }\phi \left(z\left(\omega \right)\right)\;dP\left(\omega \right)\right)+\int_{\Omega }X\left(\omega \right)z\left(\omega \right)\;dP\left(\omega \right)\right\}\\ & s.t. & \int_{\Omega }z\left(\omega \right)\;dP\left(\omega \right)=1\\ & & \alpha \leq z(\omega )\leq \beta \end{array}$$

The Lagrangian dual is given by

$$\begin{array}{ccc} w & := & \underset{\eta \in R}{\sup}\left\{\eta +\underset{\alpha \leq z(\cdot )\leq \beta }{\inf}\left\{h\left(\int_{\Omega }\phi \left(z\left(\omega \right)\right)\;dP\left(\omega \right)\right)-\int_{\Omega }\left(\eta -X\left(\omega \right)\right)z\left(\omega \right)\;dP\left(\omega \right)\right\}\right\}. \end{array}$$

If h(·) is concave, denoted by $h_{c}$ (e.g., log function), by using Jensen inequality and Lemma A1, it follows that

$$\begin{array}{ccc} w & \leq & \underset{\eta \in R}{\sup}\left\{\eta +\underset{\alpha \leq z(\cdot )\leq \beta }{\inf}\left\{\int_{\Omega }h_{c}\left(\phi (z(\omega ))\right)\;dP\left(\omega \right)-\int_{\Omega }\left(\eta -X\left(\omega \right)\right)z\left(\omega \right)\;dP\left(\omega \right)\right\}\right\}\\ & = & \underset{\eta \in R}{\sup}\left\{\eta -E_{P}\phi_{h}^{*}\left(\eta -X\right)\right\}. \end{array}$$

By applying Lemma A2, the equation is equal to the convex risk measure of the OCE-type form

$$-\rho_{OCE}^{h}\left(X\right)=\underset{Q\in Q}{\inf}\left\{E_{Q}\left[X\right]+D_{\phi_{h}}\left(Q,P\right)\right\}=\underset{\eta \in R}{\sup}\left\{\eta -E_{P}\phi_{h}^{*}\left(\eta -X\right)\right\},$$

where $\phi_{h}^{*}$ is the conjugate of $\phi_{h}:=h\circ \phi$. It completes the proof by verifying that the ‘constraint qualification’ stated in Theorem 4.2 of [8] holds, which results in w=v. For convex *h*, the proof is similar. □

**Remark** **4.** *If h is convex, the lower bound of any (h,ϕ)-divergence risk measure is its related ϕ-divergence risk measure. However, when h is concave, there may be a potential range of cases for meeting the demands from the industries since they prefer both convex and smaller measures.*

**Remark** **5.** *As the (h,ϕ)-divergence risk measure extends from a general divergence, the degree of complexity of its sensitivity analysis increases, adapting to multi-parameter forms, such as, for instance, Sharma-Mittal divergence [41,42], rather than the one-parameter cases of Tsallis- or Rényi-divergence risk measures. To handle the sensitivity analysis in relation to the multiple parameters involved, with or without different units, we refer to the framework of the differential importance measure (see [43,44] and references therein for details).*

**Rényi-divergence risk measure.** Intuitively, the Rényi-divergence risk measure is derived as

$$\rho_{R}\left(X\right)=\underset{Q\in Q}{\sup}\left\{E_{Q}\left[-X\right]-D^{R}\left(Q,P\right)\right\},$$

where

$$D^{R}\left(Q,P\right)=\frac{1}{\alpha -1}\ln\int_{\Omega }{\left(\frac{dQ}{dP}\right)}^{\alpha -1}dQ=h\left(\int_{\Omega }\phi \left(\frac{dQ}{dP}\right)\;dP\right),$$

with $h\left(x\right)=\frac{1}{\alpha -1}\ln\left(1+\ell \cdot x\right)$ and $\phi \left(x\right)=\ell \cdot \left(x^{\alpha }-1\right)$, with ℓ=sgn(α−1) and α∈(0,1). When α=1, Rényi divergence (as well as Tsallis divergence) is equal to KL divergence.

**Remark** **6.** *The parameter α of $\rho_{R}\left(X\right)$ or $\rho_{T}\left(X\right)$, as well as θ in $\rho_{M}\left(X\right)$, can be interpreted as calibrating parameters of the level of ambiguity aversion in the preferences. However, θ essentially states the balance between the expected risk and the ambiguity aversion, yet α determines the structural profile of ambiguity, which corresponds to the nature of the preferences in the decision-making agents. Their behaviors are shown in the design of the simulation in the next section. The Rényi-divergence risk measure, among others, is deeply discussed in the recent paper [45].*

It is easy to check that Rényi divergence is a convex measure of risk when α∈(0,1). Then, the lower bound of the Rényi-divergence risk measure via the convexity of *h* is

$$\rho_{R}\left(X\right)\geq \rho_{OCE}^{R}\left(X\right)=\underset{\eta \in R}{\sup}\left\{\eta -E_{P}\phi_{h}^{*}\left(\eta -X\right)\right\},$$

where

$$\begin{array}{ccc} \phi_{h}^{*}\left(x\right) & = & \underset{t\in R}{\sup}\left\{t\cdot x-h\circ \phi \left(t\right)\right\}\\ & = & \underset{t\in R}{\sup}\left\{t\cdot x-\ln\left(t^{\alpha }\right)/\left(1-\alpha \right)\right\}. \end{array}$$

## 5. Simulated Examples

The aim of the simulations described in this section is to compare the performances for the different divergence-based risk measures. One comparison is given between Rényi-divergence risk measure and Tsallis-divergence risk measure when α∈(0,1]. In both divergences, which constitute an important reference in information theory and its multiple applications [46], the parameter α calibrates the structural deviations between two probability measures and hence, as mentioned before, the profile of ambiguity aversion by the agent in the context of preferences. The second comparison is addressed to assess the effect derived in Tsallis-divergence risk measure by the extra distortion of a non-linear or linear function *h*. In this case, the parameter α is in (0,∞).

In the setting of the dual representation of convex measures of risk, it is difficult to perform numerical studies depending on the nature of the measure *P* and the supremum of *Q* without the quantified OCE-type expression. Therefore, it is natural to consider the simulation via the idea of perturbation in the compositional data analysis. We assume an initial discrete distribution $p_{i}$ of the financial position $x_{i}$, and the optimized distribution $q_{i}$ for i=1,…,n, where *n* denotes the size of the sample space, such that $\sum_{i=1}^{n}p_{i}=1$, $\sum_{i=1}^{n}q_{i}=1$. In this case, our proposed risk measure can be rewritten by the form

$$\begin{array}{cc} \rho_{h}\left(X\right)= & \max_{q_{i}}\left\{-\sum_{i=1}^{n}x_{i}p_{i}-h\left(\sum_{i=1}^{n}p_{i}\phi \left(q_{i}/p_{i}\right)\right)\right\}\\ & s.t.\;\sum_{i=1}^{n}q_{i}=1. \end{array}$$

**Figure 1:**
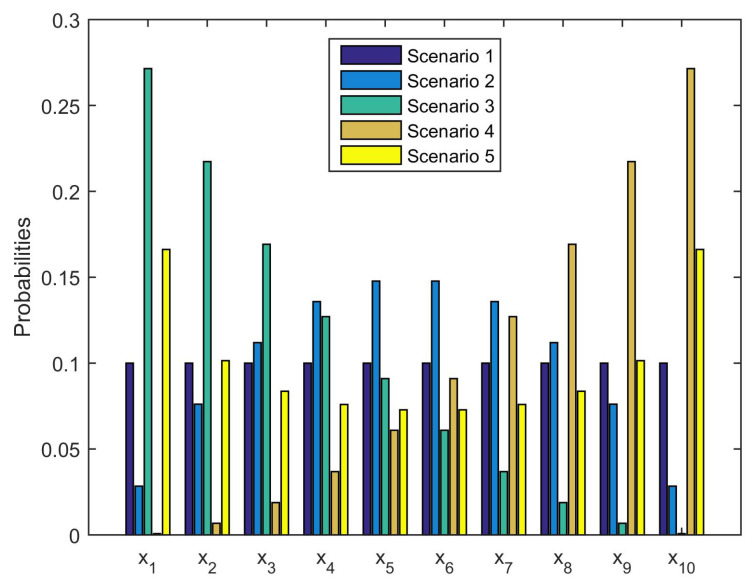
The setting of five scenarios on the reference probability measure *P*.

The complexity of this form increases rapidly with the size of the sample space, so we simplify the setting that *n* is 10 and $x_{i}$ is from 0.1 to 1 in the numerical studies. Since the influence of the reference distribution $p_{i}$ is obviously important to the value of risk measure, we took five scenarios on $p_{i}$ with respect to $x_{i}$ for consideration, see Figure 1. Scenario 1 is chosen to be the equiprobability case, $p_{i}=1/n$; Scenario 2 consists in assigning the larger probability to the value of $x_{i}$ in the middle range and the smaller probability to the small and large values of $x_{i}$; Scenario 3 is putting large probabilities on the small-value financial positions; Scenario 4 is the reverse of Scenario 3; Scenario 5 is the reverse of Scenario 2.

**Figure 2:**
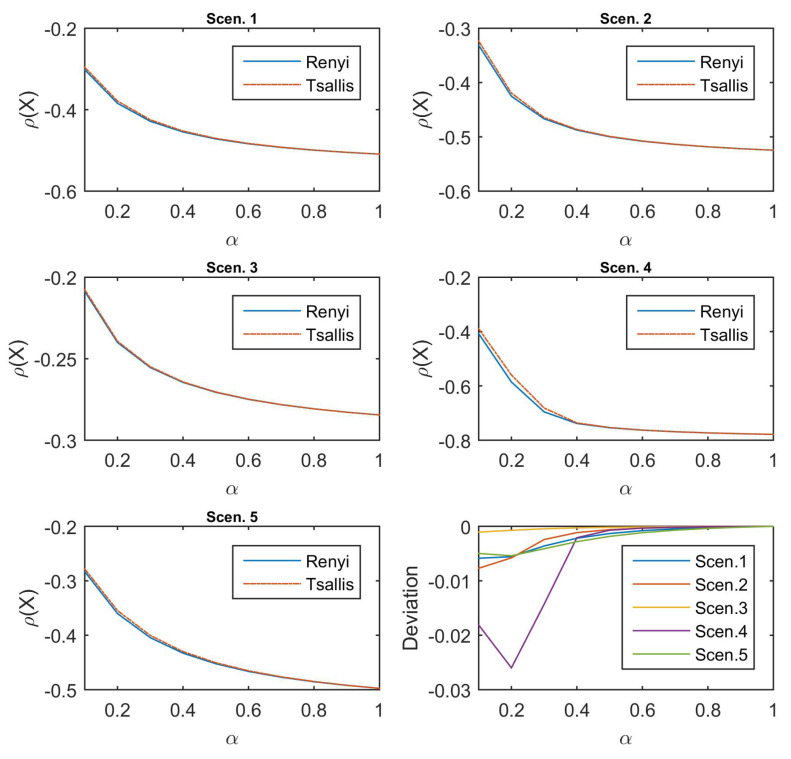
Values of Rényi-divergence and Tsallis-divergence risk measures for the different scenarios and their deviations.

Figure 2 depicts the performance of Rényi-divergence risk measure (the solid line in blue) and Tsallis-divergence risk measure (the solid line in red) under the five scenarios considered, with α∈(0,1]. All the five subfigures show the same declining shape of the values for these two risk measures for increasing α, hence the structures of the two penalties do not change that much in the measure of risk according to the same α; the value of Rényi-divergence risk measure is slightly smaller than that of Tsallis-divergence in the last subfigure of Figure 2. In addition, the deviation in Scenario 4, which puts the larger probabilities on the larger values, is smaller than in other scenarios when α is small, whereas Scenario 3, which is the reverse of Scenario 4, shows almost no deviation of both measures of risk.

**Figure 3:**
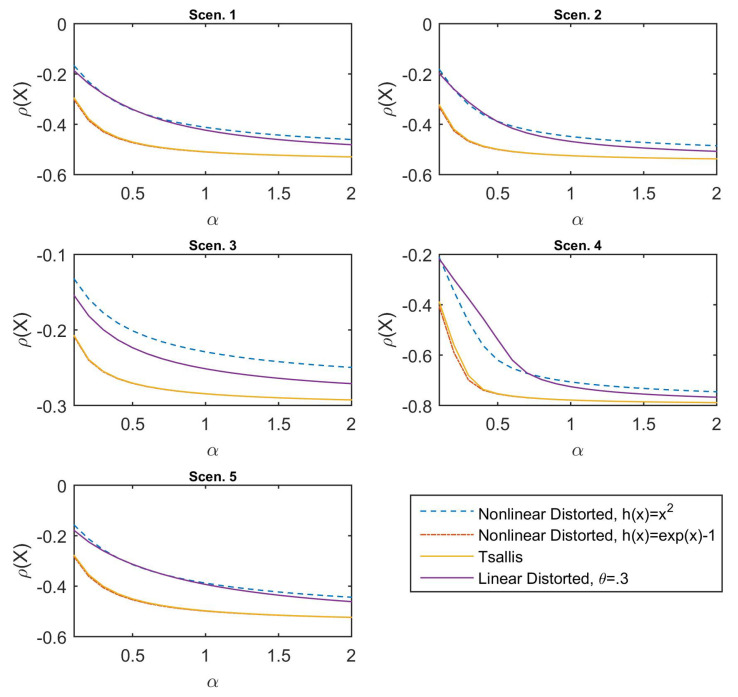
Values of Tsallis-divergence risk measure and corresponding nonlinearly and linearly distorted risk measures for the different scenarios.

For the second comparison, we consider these three non-linear and linear deformations on Tsallis divergence: (1) Second-order polynomial $h\left(x\right)=x^{2}$; (2) exponential $h\left(x\right)=e^{x}-1$; and (3) linear case θ=0.3. The results are presented in Figure 3. It can be observed that the Tsallis-family risk measures exhibit the same behavior on the declining shapes, the values of which are decreasing for increasing α. In particular, the value of the second-order polynomial (the dash line in blue) is apparently larger but declines more gently than that of the original Tsallis-divergence risk measure (the solid line in yellow), and it also leads to a much stronger distortion on the scale than that of the exponential deformation (the dash–dot line in red), the value of which is slightly smaller than the Tsallis-divergence risk measure. Moreover, even for the linear function (the solid line in purple) operating on Tsallis divergence, the risk measure reveals non-linear variations. Shortly, all the figures verify the fact that the divergence-based risk measure remains sensitive on the reference distribution of the financial positions.

## 6. Conclusions

A basic and straightforward approach to the analysis of the sensitivity of the modified ϕ-divergence convex risk measure based on Gâteaux differentiability is proposed, and further, by non-linearly distorting the ϕ divergence, a wider range of the divergence family of convex risk measures is explored. In particular, interest is focused on the deviations of the risk measure derived from slight modifications of both the input financial position in X and the probability measure in M, benefiting from the preference and robust-statistics area shared with the relative formation and frame, respectively. The extension introduced, called (*h*,ϕ)-divergence risk measure, which is inspired by the dual representation of convex risk measures and covers a larger variety of divergences as penalty functionals, also nurtures the divergence preference on expanding the class of the ambiguity attitudes. A lower bound for the (*h*,ϕ)-divergence risk measure is established in the case where *h* is a convex function, in terms of the related (h∘ϕ)-divergence risk measure.

Several directions for study on the (*h*,ϕ)-divergence risk measure are open and are beyond the sensitivity and boundedness properties discussed in this paper. First, the relationship with the certainty equivalent, in a similar way to the general Donsker-Varadhan formula or the relation between ϕ-divergence risk measure and OCE, would be useful in order to efficiently implement the quantification of risk in practical applications. In this case, the sensitivity property of the (*h*,ϕ)-divergence risk measure, with respect to the reference probability, can also be studied through the relation. Furthermore, as shown in [39,40], the framework of risk measures on Orlicz space introduced by [47], applying merely to the law-invariant convex risk measures, is useful for studying their qualitative robustness through Kusuoka representations. Exploring the specific type and comparative degree of robustness for general convex risk measures defined through the dual representation, such as the ϕ-divergence or (h,ϕ)-divergence risk measures, also constitutes an important challenge of interest for continuing research.

## Appendix A.

### Appendix A.1. Preliminary and Setup

This section follows the settings of [8] in the space $L^{p}\left(\Omega ,F,P\right)$ and its dual space. Let (Ω,F) be a measurable space equipped with σ-algebra F. Consider two probability distributions *P* and *Q*, and let μ be an arbitrary dominating positive measure of *P* and *Q*, such that both *P* and *Q* are absolutely continuous with respect to μ on F, which we denote by P≪μ, Q≪μ. Following the Radon-Nikodym theorem, there exist F-measurable functions x,y≥0 representing the densities of *P* and *Q* with respect to μ, respectively, written as

$$x=\frac{dP}{d\mu },\;y=\frac{dQ}{d\mu }.$$

For 1≤p≤+∞, let $L^{p}:=L^{p}\left(\Omega ,F,P\right)$ be the linear space of measurable real-valued functions f:Ω→R with ${∥f∥}_{p}<\infty$. For p∈[1,+∞), we denote by $L^{q}$ its dual space, with q∈(1,+∞] and p+q=qp. Furthermore, for $y\in L^{p}$ and $X\in L^{q}$,

$$<y,X>=\int_{\Omega }y\left(\omega \right)X\left(\omega \right)\;d\mu \left(\omega \right)=\int_{\Omega }X\left(\omega \right)\;dQ\left(\omega \right)=E_{Q}\left[X\right],$$

and this partially leads to the Legendre-Fenchel form for the risk measure.

### Appendix A.2. Related Results

Let ϕ:R→(−∞,∞] be a proper closed convex function such that domϕ is an interval with endpoints a<b, thus int domϕ=(a,b). Since ϕ is closed,

$$\lim_{t\to a+}\phi \left(t\right)=\phi \left(a\right),\lim_{t\to b-}\phi \left(t\right)=\phi \left(b\right)$$

if *a* and *b* are finite. It is assumed that 1∈int domϕ and that the minimum of ϕ is 0. The class of such functions is denoted by Φ.

**Definition** **A1** (ϕ-divergence)**.**
*Given ϕ∈Φ, the ϕ-divergence of the probability measure Q with respect to P is*

$$D_{\phi }\left(Q,P\right)=\int_{\Omega }\phi \left(\frac{dQ}{dP}\right)\;dP,\;ifQ\ll P;$$

*otherwise, $D_{\phi }\left(Q,P\right)=+\infty$.*

To avoid some meaningless cases, it is assumed that

$$\phi \left(0\right)<\infty ;\;0\phi \left(\frac{0}{0}\right)=0;\;0\phi \left(\frac{s}{0}\right)=\lim_{\varepsilon \to 0}\varepsilon \phi \left(\frac{s}{\varepsilon }\right)=s\lim_{t\to +\infty }\frac{\phi (t)}{t},\;s>0.$$

**Definition** **A2** (Legendre-Fenchel transform)**.**
*Let ϕ∈Φ. The conjugate of a real-valued function ϕ on $R^{d}$ is another real-valued function on $R^{d}$, defined as*

$$\phi^{*}\left(x\right):=\underset{t\in R^{d}}{\sup}\left\{t\cdot x-\phi \left(t\right)\right\}.$$

When d=1, the conjugate $\phi^{*}$ is a closed proper convex function, with $int dom\phi^{*}=\left(a^{*},b^{*}\right)$, where

$$a^{*}=\lim_{t\to -\infty }t^{-1}\phi \left(t\right)\in \left[-\infty ,+\infty \right),\;b^{*}=\lim_{t\to +\infty }t^{-1}\phi \left(t\right)\in \left(-\infty ,+\infty \right].$$

**Lemma** **A1** (The interchange of minimization and integration)**.**
*Let Ω be a σ-finite measure space, and let $X:=L^{p}\left(\Omega ,F,P\right)$, p∈[1,+∞]. Let g:R×Ω→(−∞,+∞] be a normal integrand. Then,*

(A1) $$\underset{x\in X}{\inf}\int_{\Omega }g\left(x\left(\omega \right),\omega \right)\;dP\left(\omega \right)=\int_{\Omega }\underset{s\in R}{\inf}g\left(s,\omega \right)\;dP\left(\omega \right).$$

**Lemma** **A2** (The generalized Donsker-Varadhan variational formula)**.**
*Let ϕ:R→(−∞,+∞] be a closed convex function with g(0)=0. Then, for $X\in L_{\infty }$,*

$$\underset{Q\in Q}{\inf}\left\{E_{Q}\left[X\right]+D_{\phi }\left(Q,P\right)\right\}=\underset{\eta \in R}{\sup}\left\{\eta -E_{P}\phi^{*}\left(\eta -X\right)\right\},$$

*where $\phi^{*}$ denotes the conjugate of ϕ via the Legendre-Fenchel transform. In particular, for ϕ(x)=xlnx−x+1 if x≥0 and ϕ(x)=∞ otherwise, it retrieves the classical Donsker-Varadhan variational formula presented in the Large Deviation Theory.*

**Lemma** **A3** (Varadhan’s lemma)**.**
*Suppose that $\left\{X^{\varepsilon }\right\}$ satisfies a large deviation principle on X with good rate function I. Then, for any bounded continuous function φ:X→R, we have*

$$\lim_{\varepsilon \to 0}\varepsilon \ln E\left[e^{\varphi (X^{\varepsilon })/\varepsilon }\right]=\underset{x\in X}{\sup}\left[\varphi \left(x\right)-I\left(x\right)\right].$$
